# Ipecacuanha in Emetic Doses, as a Powerful Restorative in Some Cases of Exhaustion and Sinking

**Published:** 1845-09

**Authors:** John Higginbottam

**Affiliations:** Nottingham


*Ipecacuanha, in Emetic Doses, as a powerful Restorative in some cases of Exhaustion and Sinking.* By John Higginbottam, F.R.C.S., Nottingham. (Read before the Nottingham Medico-Chirurgical Society, May 23, 1845.)

In the year 1814, I was first led to see the extraordinary beneficial effects of ipecacuanha, as an emetic, in a female forty years of age, who was in a sinking state, in the last stage of cholera; her countenance was shrunk, extremities cold, cramp in the legs, and other symptoms of approaching dissolution. I had previously attended two similar cases, where I had given opium, brandy, and medicinal cordials, and both patients died. I was induced, in this instance, to give a scruple of ipecacuanha, from having frequently seen the good effects of it in the early stage of the disease. After the lapse of two or three hours, I again visited my patient, fearing I should find her dead, but, to my great pleasure and surprise, so great a change for the better had taken place as to appear almost incredible; the whole of her body was of a natural warmth, the dangerous symptoms had disappeared, and she made no complaint, except that she was very weak. She had no further unfavorable symptom of the disease, and was soon convalescent.

My confidence in the ipecacuanha, as a remedy in such cases, has now been confirmed during the practice of thirty years; the purging, vomiting, and cramp, often entirely cease after the emetic operation of the ipecacuanha, but I have thought it proper to give, in about two or three hours after the emetic, a pill, with a grain of opium and five grains of the blue pill, to allay any remaining irritation of the stomach and intestines and an aperient, with one scruple of rhubarb and two of the sulphate of potash, to assist the natural action of the bowels, and a simple saline effervescing draught every two or three hours afterwards: weak tea, well-boiled gruel, milk, with sago or arrow-root as nutriment, and diluents.—*Dub. Med. Press.*

